# Bird Species Involved in West Nile Virus Epidemiological Cycle in Southern Québec

**DOI:** 10.3390/ijerph17124517

**Published:** 2020-06-23

**Authors:** Ludivine Taieb, Antoinette Ludwig, Nick H. Ogden, Robbin L. Lindsay, Mahmood Iranpour, Carl A. Gagnon, Dominique J. Bicout

**Affiliations:** 1 Research Group on Epidemiology of Zoonoses and Public Health (GREZOSP), Faculty of Veterinary Medicine, Université de Montréal, Saint-Hyacinthe, QC J2S 2M2, Canada; ludivine.taieb@gmail.com (L.T.); nicholas.ogden@canada.ca (N.H.O.); 2 Department of Pathology and Microbiology, Faculty of Veterinary Medicine, Université de Montréal, Saint-Hyacinthe, QC J2S 2M2, Canada; carl.a.gagnon@umontreal.ca; 3 Public Health Risk Sciences Division, National Microbiology Laboratory, Public Health Agency of Canada (PHAC), Saint-Hyacinthe, QC J2S 2M2, Canada; 4 Zoonotic Diseases and Special Pathogens Division, National Microbiology Laboratory, Public Health Agency of Canada (PHAC), Winnipeg, MB R3E 3R2, Canada; robbin.lindsay@canada.ca (R.L.L.); mahmood.iranpour@canada.ca (M.I.); 5 Swine and Poultry Infectious Diseases Research Center (CRIPA), Faculty of Veterinary Medicine, Université de Montréal, Saint-Hyacinthe, QC J2S 2M2, Canada; 6 Biomathématiques et Épidémiologie, EPSP-TIMC, UMR CNRS 5525, Université Grenoble-Alpes, VetAgro Sup, 38700 La Tronche, France; 7 Laue-Langevin Institute, Theory Group, 71 Avenue des Martyrs, 38042 Grenoble, France

**Keywords:** West Nile, wild birds, *Culex* feeding/host preference, eco-epidemiology, Québec

## Abstract

Despite many studies on West Nile Virus (WNV) in the US, including the reservoir role of bird species and the summer shifts of the *Culex* mosquito, feeding from birds to mammals, there have been few equivalent studies in the neighboring regions of Canada where WNV is endemic. Here, a priority list of bird species likely involved in WNV transmission in the greater Montréal area is constructed by combining three sources of data: (i) from WNV surveillance in wild birds (2002–2015); (ii) blood meal analysis of *Culex pipiens–restuans* (CPR), the primary enzootic vectors of WNV in the region, collected from surveillance in 2008 and 2014; (iii) literature review on the sero-prevalence/host competence of resident birds. Each of these data sources yielded 18, 23 and 53 species, and overall, 67 different bird species were identified as potential WNV amplifiers/reservoirs. Of those identified from CPR blood meals, Common starlings, American robins, Song sparrows and House sparrows ranked the highest and blood meal analysis demonstrated a seasonal shift in feed preference from birds to mammals by CPR. Our study indicates that there are broad similarities in the ecology of WNV between our region and the northeastern US, although the relative importance of bird species varies somewhat between regions.

## 1. Introduction

**Figure 1:**
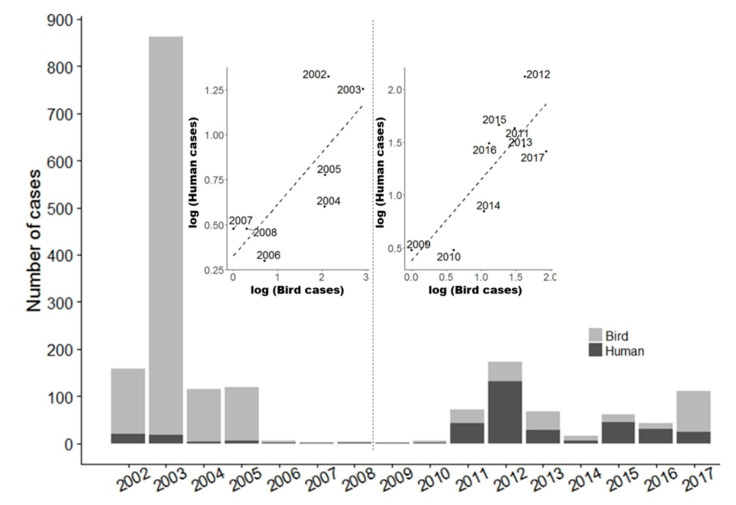
Yearly number of cases of West Nile virus (WNV) infection in humans and number of dead birds positive to WNV in Québec 2002–2017. Insert: Correlation between the number of WNV bird and human cases in Québec.

First described in Uganda in 1937 [1], West Nile Virus (WNV) is an arbovirus of the *Flaviviridae* virus family, genus *Flavivirus*. It has a transmission cycle involving mosquitoes as vectors and birds as amplifying hosts or reservoirs, with humans and horses being primarily dead-end hosts [2,3]. In humans, the early symptoms of WNV include fever, headache, skin rash, nausea and muscle aches. Most of the affected people recover fully, but approximately 1% develops severe illness (meningitis, encephalitis, acute flaccid paralysis and poliomyelitis) [4]. Those over 70 years of age with underlying medical conditions and those who are immuno-compromised are at a greater risk of severe illness [4]. In North America, the first WNV outbreak occurred in New York in 1999, and WNV spread rapidly across the continent, causing mortality in many bird species [5,6,7]. In less than four years, the virus spread to most of the continental United States, and its activity was first reported in Canada in 2001 [8]. WNV is now endemic in much of southern Canada and, given the potential severity of the disease in humans and the lack of treatment or a specific vaccine, WNV infection is a major public health concern in Canada, including in the province of Québec [9]. Most human cases of WNV reported in Canada occur in Québec, Ontario and the Prairie provinces, and the intensities of the outbreaks of WNV vary temporarily and spatially, as shown in Figure 1. In Québec, human cases of WNV are mainly reported from the Montérégie, Montréal and Laval regions of southern Québec [9]. This study focuses on the Montréal area, one of the regions most affected by WNV in Québec, with 28% of the clinical cases of WNV reported between 2002 and 2014 [9].

The epizootiological cycle of WNV transmission involves bird of several species that act as reservoirs/amplifying hosts and a range of mosquito species that transmit the virus amongst animals (enzootic vectors) and/or to humans (epizootic vectors). Mammals, such as horses and humans, are incidental or dead-end hosts that are not part of the virus transmission cycle because they do not produce viremia sufficient to infect mosquitoes [10]. In southeastern Canada, including Québec, the main WNV enzootic vectors are *Culex pipiens* and *Culex restuans* [11] and these species are also involved in the transmission of WNV to humans in neighboring parts of the northeastern US [12,13,14].

A range of bird species can act as reservoir hosts for WNV, and annual migration by many species disperses WNV over long distances [15,16,17,18,19]. The importance of birds species as amplifying hosts depends on a combination of factors: (i) the susceptibility to infection; (ii) the duration of viremia at levels high enough to infect feeding mosquitoes; (iii) the density of naïve individuals (a combination of the density of the species and rates of infection followed by protective immunity); (iv) the “attractiveness” of the species to (ornithophilic and opportunistic) mosquito vectors and thus the proportion of mosquito bites per unit of space–time that occur on the species; and, (v) the rates of mortality, including WNV-specific mortality, of infected individuals.

Experimental studies have shown that several North American bird species are susceptible to WNV and can transmit the virus because they produce a level of viremia that is sufficient to infect mosquitoes that feed upon them—some species die as a result of this infection [20]. Mortality in wild bird populations, particularly corvids, was used as an early surveillance signal of WNV activity in a given locality, as the virus first spread across the US and then Canada [21,22]. Wild bird mortality was also used as an index of the rates of expected human cases of WNV [23]. Retrospective analysis suggested that, when it first invaded North America, WNV caused mortality in a wide range of bird species [24].

Studies in the US have taken into account the multiple factors that define reservoir competence, and by combining field observations and laboratory experiment results, they conclude that the American robin is a key reservoir species [12]. Furthermore, studies from the US suggest that the seasonal nature of human WNV cases (with most cases from late summer through to mid-autumn) is associated with a shift in mosquito blood meals from birds to mammals during the high-risk period, which may be driven by birds beginning their southward migration at this time [13]. To date, similar studies on the transmission dynamics of WNV in Canada are lacking.

In this study, we aimed to develop a priority list of bird species likely involved in the circulation of WNV in the region of Montréal in southern Québec, Canada. To do so we scanned the literature and dead bird surveillance data to identify bird species that breed in the region and are known to be competent WNV amplifiers/reservoirs. We also prioritized these species according to field and laboratory data on the feeding preferences of *Culex pipiens–restuans* (CPR) mosquitoes, while accounting for bird species abundance in the Montréal area. The blood meal analysis data also allowed us to explore the occurrence of seasonal shifts in the host-feeding behavior of key species of vector mosquitoes. Collectively this data allowed us to determine the bird species that are key amplifier/reservoir hosts for WNV and to determine the extent to which host shifting occurs in vector mosquitoes in Québec.

## 2. Materials and Methods

### 2.1. Study Area

**Figure 2:**
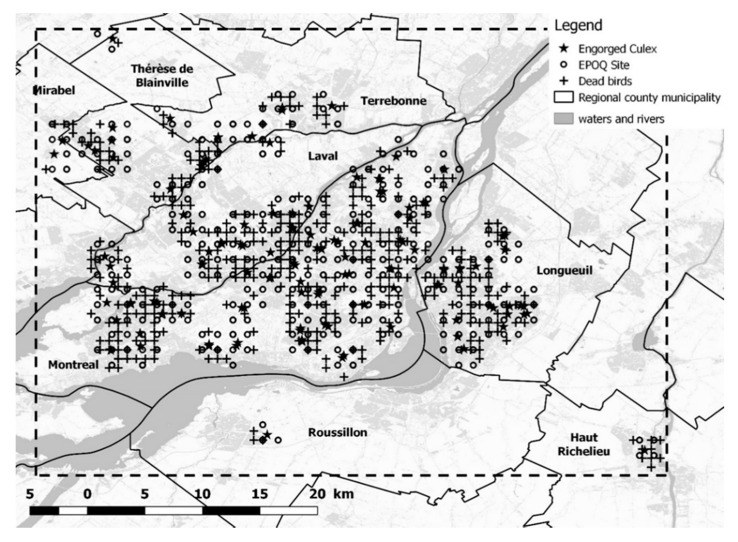
Study area with locations of collection of dead birds (CWHC), bird watching and counts (EPOQ) and collection of engorged female *Culex pipiens–restuans* on the island of Montréal, Québec.

The greater Montréal area is located in the southern Québec, as shown in Figure 2, and in the central part of the St. Lawrence Lowlands. The region is bordered to the north by the Canadian Shield and to the southeast by the Appalachians [25]. The region is characterized by a temperate continental climate, with cold winters and hot summers [25]. The study area is 30,231.2 km^2^, and includes the island of Montréal and was limited by the coordinates 46°13’48 N, 45°1’12 N and 74°24’36 W and 72°23’24 W. In 2011, the greater Montréal area was comprised of 21% buildings, 40% cultivated agricultural land and 39% natural or semi-natural areas, of which 66% was forest [26]. With 2,900,000 inhabitants, about 45% of the Québec province’s population, the greater Montréal area is the most densely populated area in Québec and the second most densely populated city in Canada.

### 2.2. Identification of Priority List of Wild Bird Reservoir Species

To construct the list of bird species that are possible WNV amplifier and reservoir host in our study area, we used an approach by successive augmentation. Starting with a reference list of regional breeding bird species $L_{0}$, the priority list $L_{f}$ of birds that could potentially play a role in the WNV transmission cycle was obtained as, $L_{f}=L_{0}\cap^{\;}\left[L_{1}+L_{2}+L_{3}-L_{1}\cap^{\;}L_{2}-L_{1}\cap^{\;}L_{3}-L_{2}\cap^{\;}L_{3}+L_{1}\cap^{\;}L_{2}\cap^{\;}L_{3}\right]$, where $\cup^{\;}$ indicates the “union” of data from databases (bringing all species from the databases together), and $\cap^{\;}$ indicates the “intersection” of data from the databases (i.e., where species are in the different databases). $L_{1}$, $L_{2}$ and $L_{3}$ are, respectively, the “databases” of: (i) species identified as infected by WNV in dead bird surveillance; (ii) species identified as being targets for *CPR* blood meals on the basis of blood meal analyses; (iii) species identified as WNV reservoirs by literature review. In summary, $L_{1}$ is the first basic list that is augmented with species from $L_{2}$ and $L_{3}$ not already in $L_{1}$. The comparison with $L_{0}$ is a check to ensure that selected bird species are found in the study area. $L_{0}$ comprised 318 species extracted from the Avibase database [27], which includes all breeding species in the Montréal area. Species reported as rare (*n* = 65) in the Avibase were excluded from $L_{0}$. Details on $L_{1}$, $L_{2}$ and $L_{3}$ are described in the sub-sections below.

#### 2.2.1. Wild Bird Mortality Data: List $L_{1}$

Wild bird mortality data were obtained from the Canadian Wildlife Health Cooperative (CWHC) Passive Mortality Monitoring Program [28], and data specific to WNV mortality were acquired from the passive WNV wild bird surveillance program, which was established in 2001, the first year that WNV was detected in Canada [29]. This “passive” surveillance work was carried out in collaboration with the local population, which was invited to report dead bird sights to the relevant authorities. Dead birds were retrieved and submitted to veterinary diagnostic laboratories [29]. In these laboratories, necropsies were performed, and selected tissues were tested for WNV [29,30]. The data cover the period 2002–2015, during which time two WNV epidemics occurred in Québec. The locations where dead WNV-infected birds were collected are reported in Figure 2. To rank birds species belonging to $L_{1}$, the standardized mortality ratio, or relative ratio, $RR_{s}$, for each bird species “s” was calculated as, $RR_{s}=m_{s}/\left(\lambda \times n_{s}\right)$, where $m_{s}$ is the number of dead birds of species “s” found positive for WNV, $n_{s}$ is the corresponding sample size and $\lambda =\underset{s}{\sum }m_{s}/\underset{s}{\sum }n_{s}$, is the mortality rate under the homogeneous hypothesis; $\left(\lambda \times n_{s}\right)$ is the expected number of dead birds of species “s”.

#### 2.2.2. Blood Meal Data: List $L_{2}$

Mosquitoes were collected in our study region between 2008 and 2014 as part of a provincial mosquito surveillance program conducted in southern Québec [9]. Blood meal analysis was conducted only on engorged females of either the *Culex pipiens* or *Cx. restuans* (*CPR)* complex in this study. The capture sites of engorged females are shown in Figure 2. The list $L_{2}$ consists of bird species identified as blood meal sources for *CPR* complex mosquitoes. Note that females of *Culex pipiens* and *Cx. restuans* cannot be reliably differentiated morphologically, so the specimens were grouped together as *CPR* complex mosquitoes.

Blood-fed mosquitoes were stored individually in 1.5 mL tubes at −80 °C until processing. The extraction of DNA was carried out using a protocol described by Molaei et al. [31]. Briefly, 200 µl of DNAzol^®^ BD (Molecular Research Center, Cincinnati, OH, USA) was added to each tube. The mosquitoes were homogenized with a pestle, followed by the addition of another 200 µL DNAzol BD and 15 µL proteinase-K. The tubes were vortexed briefly, incubated at 70 °C for ten minutes, then centrifuged for ten minutes at 14,000 rpm. The supernatants were transferred to new 1.5 mL tubes and 3 µL polyacryl carrier (Molecular Research Center, Cincinnati, OH, USA) was added to each tube. The tubes were incubated at room temperature for three minutes and then 200 µL 100% ethanol was added. Following mixing by gentle inversion, the tubes were incubated on ice for ten minutes, then centrifuged for six minutes at 6000 rpm. The supernatants were removed, and the remaining DNA pellets were washed twice with the addition of 750 µL 75% ethanol and two minutes of centrifugation at 3000 rpm. After the final removal of the ethanol, the tubes were left open to allow the DNA pellets to air dry. Once dry, the pellets were re-suspended in 20 to 50 µL 1 × TE buffer. The DNA extracts obtained from blood-fed mosquitoes were used as templates for the amplification of the *cytochrome b* gene in avian and mammalian species using primers previously described by Molaei et al. [31]. The extracted DNA was amplified in 50 µL reactions using the Platinum taq DNA polymerase system (Invitrogen, Carlsbad, CA, USA) with final concentrations of 1.5 mM MgCl_2_, 200 nM dNTP and 200 nM per primer. Amplification was carried out in the Applied Biosystems GeneAmp PCR System 9700 using the following conditions: denaturation for two minutes at 94 °C, 40 cycles of amplification consisting of 30 s at 94 °C, 50 s at 55 °C (mammalian) or 60 °C (avian), 60 s at 72 °C, extension for seven minutes at 72 °C and then held at 4 °C. The first 100 amplification products were visualized by gel electrophoresis on 1.9% agarose gel, whereas the remaining samples were run on QIAxcel (Qiagen, Toronto, TO, Canada). Positive samples were purified using the Wizard SV Gel and PCR Clean-up system (Promega, Madison, WI, USA), and Sanger sequencing was performed by the NML Genomics Core Facility using Applied Biosystems 3730 xl DNA Analyzer with Big Dye Terminator version 3.1 and pop7 chemistries. The sequence data were analyzed using DNASTAR Lasergene 9 software and compared to those in the GenBank (NCBI). Since the technology used does not allow for the accurate identification of mixed blood meals, the sequence data from mosquitoes that took multiple host blood meals from different species did not lead to identifiable host species. However, we expected that these events would be rare because adult female *CPR* typically complete feeding on a one host.

Identified bird species (see Results section) are characterized by the proportion $f_{s}$, defined as the ratio of CPR blood meals taken from the species *s* divided by the CPR blood meals from all identified bird species. The value of $f_{s}$ depends both on the relative density of species *s* and on the degree to which a species may be particularly attractive to the mosquitoes—if so, then mosquito bites on a particular species may be disproportionate to the density of the species. To explore this, a feeding preference index can be obtained as [32]: $p_{s}=f_{s}/a_{s}$, where $f_{s}$ is as defined above and $a_{s}$ is the ratio of the abundance of species *s* over that of the total density of the birds in the area. Such a $p_{s}$ can be regarded as the relative risk for a given bird species of being bitten by the mosquito in relation to its relative abundance [32]. Unfortunately, $p_{s}$ gets larger and tends to infinity when $a_{s}$ tends to zero. Therefore, we use the following definition for the preference index [33,34]: $p_{s}=a_{s}f_{s}/\overset{n}{\underset{j=1}{\sum }}a_{j}f_{j}$, where n is the total number of bird species identified from CPR blood meals. Likewise, the relative risk for a given bird species of being bitten by the mosquito, relative to homogeneous abundance, is $RR_{s}=n\times p_{s}$. Bird species bitten more or less often than chance are characterized by $RR_{s}>1$ and $RR_{s}<1$, respectively.

Values for $a_{s}$ were obtained from wild bird count data, taken from the EPOQ-ebird database, managed, in part, by the Regroupement Québec Oiseaux (RQO) for the years 2001 to 2016 [35]. This database contains more than six million observations made by ornithologists during their daily bird watching trips within Québec. These data are obtained from opportunistic sampling—each observer records the species observed, as well as the number of individuals of each species seen. These sets of observations correspond to an observation site at a given date during a given period of time, as well as to the number of individuals of each species observed. Locations at which bird counts were made are shown in Figure 2.

##### Analysis of Seasonal Bird-to-Mammal Feeding Shift of CPR Mosquitoes

*Culex* mosquitoes are classified as ornithophilic species (i.e., mainly feeding on birds) [13,21,36]. We investigated whether the feeding preferences of the mosquitoes changed over the activity season. We considered the fraction (or probability $\pi_{\mathrm{bird}}$) of blood meals taken on bird species (i.e., number of blood meals on all bird species only/total number of blood meals over all species (birds and mammals)) as a function of the week of the year. Logistic regression was used to model the variation of the feeding preferences over weeks as follows: $\mathrm{logit}\left(\pi_{\mathrm{bird}}\right)=\beta_{0}+\beta_{1}\mathrm{week}$. Statistical analyses were performed using the GLM (generalized linear model) function in R, version 1.1.383 [37].

#### 2.2.3. Literature Review: List $L_{3}$

Literature research was conducted in August 2017 in five electronic databases: Scopus, Pubmed, CAB Abstract, Embase and Medline. The search terms used for searching in all databases were: “(West Nile Fever OR West Nile Virus) AND (Bird* OR Avian) AND (Mortality OR Sero-prevalence OR prevalence OR competence OR capacity OR transmission) AND (USA OR Canada)”. All articles published from 1 January 1999 to 16 August 2017 (end of the search) were included in the selection process using criteria on the language, title, and abstract, as shown in Table 1. In short, selected studies had to be written in English or French, dealing with an epidemiological content about bird susceptibility (host competence, WNV-induced mortality, etc.) to WNV in Canada or the United States. Data on sero-prevalence and host competence were extracted from the retrieved publications. To rank birds species belonging to $L_{3}$, the standardized WNV sero-positive ratio, or relative ratio, $RR_{s}$, for each bird species “s” was calculated as, $RR_{s}=m_{s}/\left(\lambda \times n_{s}\right)$, where $m_{s}$ is the number of WNV sero-positive birds of species “s”, $n_{s}$ is the sample size of the tested birds and $\lambda =\underset{s}{\sum }m_{s}/\underset{s}{\sum }n_{s}$ is the sero-positive rate under the homogeneous hypothesis; $\left(\lambda \times n_{s}\right)$ is the expected number of WNV sero-positive birds of species “s”.

Migratory status (migratory M, resident R birds or both M and R) and wintering and breeding areas were also added for each bird species. Wintering/breeding areas were defined as the three main regions of the East Atlantic migration corridor: region of Québec QC, North USA and South USA, delimited by the northern border of the states of North Carolina and Tennessee [38]. The two US areas make it possible to take into account short and long distance migration. The migratory status of bird species allowed us to account for the possible geographic origin of WNV infection (acquired in the wintering zone vs. breeding zone) and the possible role of each bird species in the dispersal of WNV in North America.

## 3. Results

### 3.1. Wild Bird Mortality Data: List $L_{1}$

**Figure 3:**
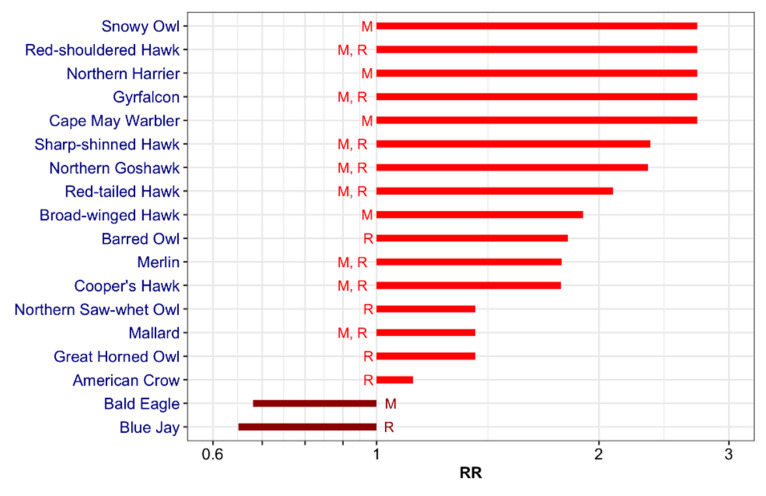
List $L_{1}$: ranked list (*n* = 18) of dead birds found by WNV passive bird surveillance. RR stands for relative risk or ratio of dead birds found positive for WNV. Quoted letters “M” and “R” stand for migratory and resident birds, respectively.

Data from the passive WNV wild bird surveillance are reported below providing Characteristics of bird species, column “Mortality”. We found a list $L_{1}=18$ that was sorted based on the relative ratio of dead birds positive for WNV, as shown in Figure 3. Of all these bird species, only two (Bald eagle and Blue jay) were under-represented (with the relative ratio of mortality < 1) in mortality data.

### 3.2. Blood Meal Analysis: List $L_{2}$

DNA from 273 engorged mosquitoes was extracted, although only 263 were included in the study as ten did not have a sufficient volume of DNA for amplification with both primer sets. In total, 97 out of 263 (36.9%) were positive using PCR and sequencing with avian primers, whereas 14 out of 263 (5.3%) were positive with PCR and sequencing using mammalian primers. Moreover, 10 out of 263 extracts (four positives with avian primers and six positives with mammalian primers) were reported as indeterminate, as sequencing of the PCR product was not successful and there was insufficient volume remaining for repeat testing, as shown in Table 2.

**Figure 4:**
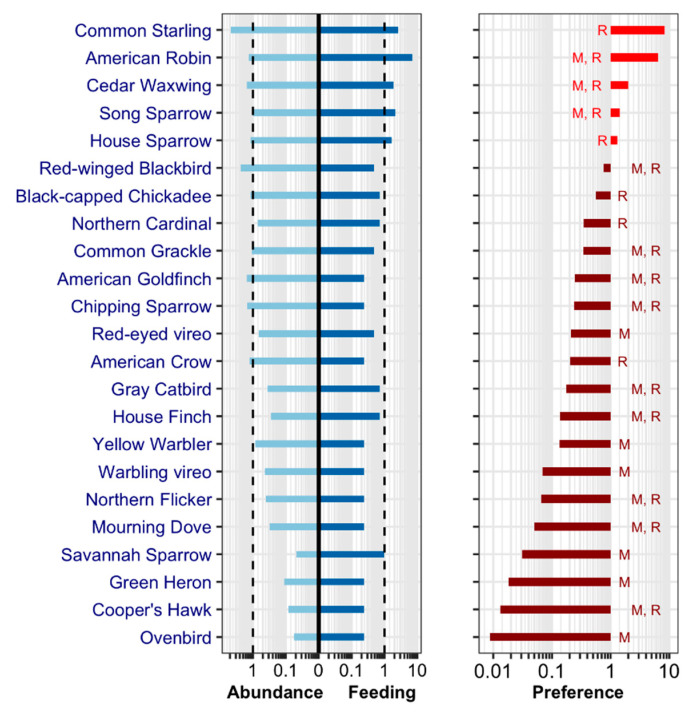
List $L_{2}$: ranked list (n = 23) of bird species found from analysis of Culex pipiens–restuans (CPR) blood meals. Left panel: Relative abundance ($n\times a_{s}$ ) of bird species and relative feeding (fraction of CPR blood meals) ($n\times f_{s}$ ). Dashed vertical lines at relative abundance and feeding “1” represent the ratio “1/*n*” where *n* = 23 is the bird species diversity. Right panel: relative host preference ($n\times p_{s}$ ) of *CPR*. Quoted letters “M” and “R” stand for migratory and resident birds, respectively.

Twenty-three ($L_{2}=23$) different bird species were identified as hosts for CPR mosquitoes, as shown in Figure 4. Most bitten bird species (with relative feeding ≥ 1) belonged to the Passeriformes order, with the American robin (31%; *n* = 30) being the most commonly identified, followed by the Common starling (11.3%; *n* = 11), the Song sparrow (9.3%; *n* = 9) and the Cedar waxwing (8.2%; *n* = 8), as shown in Figure 4 in the left panel. A large proportion of the bird species (13 out of 23) were represented by only one or two blood meals. White-tailed deer were the most bitten mammal species, while two blood meals were from humans in weeks 27 (first week of July) and 32 (first week of August). Likewise, from the data in Table 3, the most abundant birds (with relative abundance ≥ 1) were Common starling (21%) and Red-winged blackbird (10%), followed by Cedar waxwing, American goldfinch, Chipping sparrow, American robin and American crow (all with 6%), as shown in Figure 4 in the left panel.

To explore the feeding preference of CPR, the list $L_{2}$ was sorted based on the value of $p_{s}$, as shown in Figure 4 in the right panel. Of these, the highest ranked species (with relative feeding preference ≥ 1) were (descending order): Common starling, American robin, Song sparrow and House sparrow. All other bird species were associated with a relative feeding preference lower than 1, suggesting that, while very abundant (as with Red-winged blackbirds), these species are not fed upon by CPR mosquitoes, as shown in Figure 4.

#### Analysis of Seasonal Bird-To-Mammal Feeding Shift of CPR Mosquitoes

**Figure 5:**
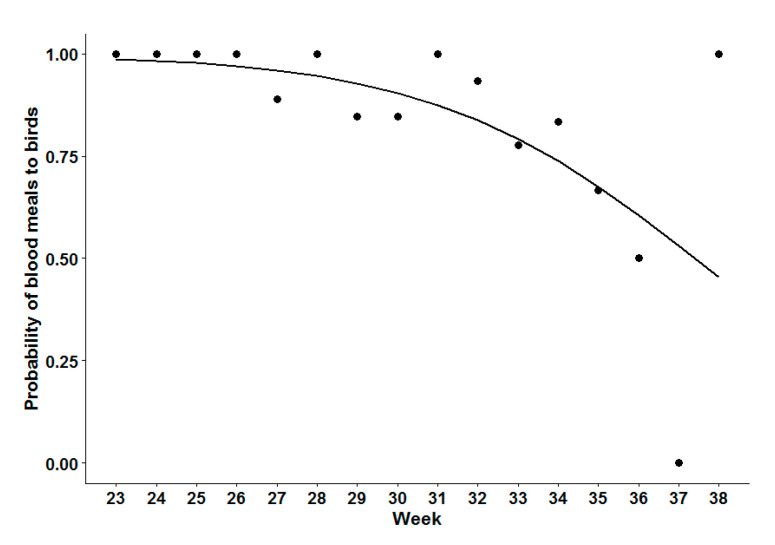
Weekly proportion of *Culex pipiens–restuans* blood meals taken from birds of all species. Data points correspond to field data and the solid line through the data corresponds to the predicted proportions using the logistic regression model.

There was a significant shift in the proportion of blood meals obtained from mammals compared to birds over the season (coefficient = −0.27 (95% confidence interval = −0.47; −0.06), *p* < 0.01). The shift appeared to be gradual and continuous from week 26 onwards, with an odds ratio of change in the proportion of blood meals from birds of 0.76 (95% confidence interval = 0.62–0.94) per week, as shown in Figure 5.

### 3.3. Literature Review: List $L_{3}$

**Figure 6:**
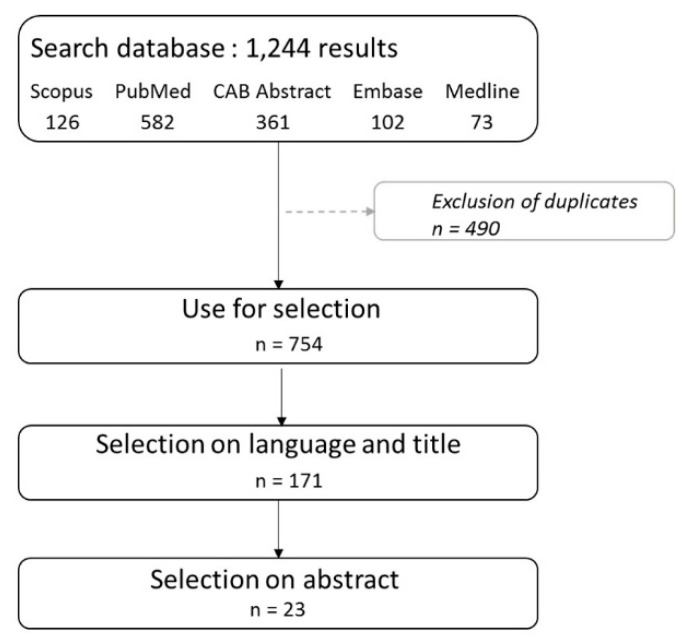
Selection process of articles and the result of the literature review.

**Figure 7:**
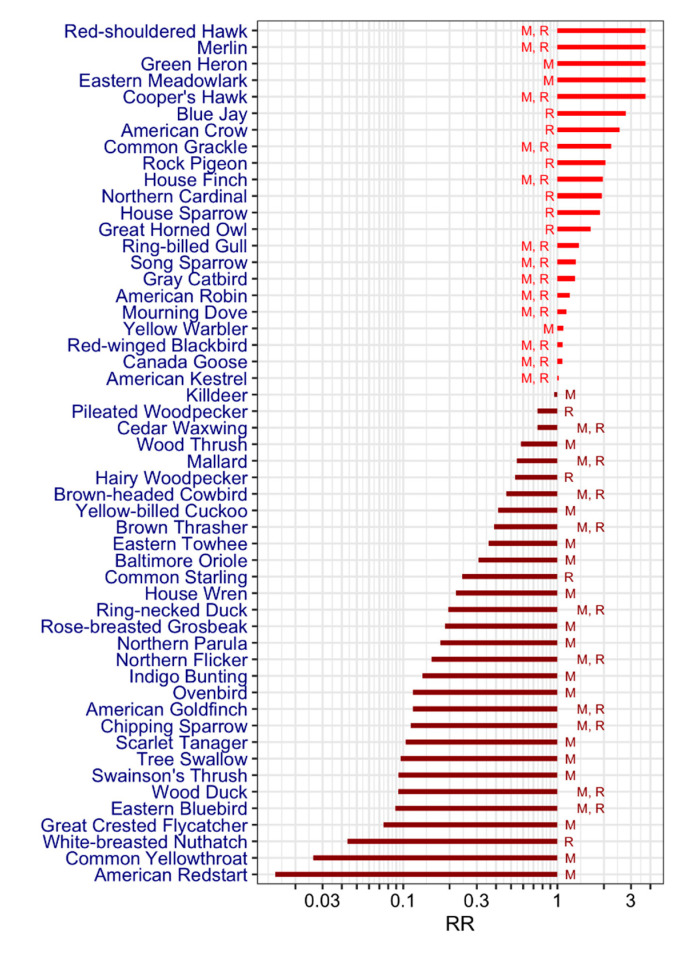
List $L_{3}$: ranked list (*n* = 53) of bird species found from the literature review. RR stands for relative risk or ratio of sero-positives. Quoted letters “M” and “R” stand for migratory and resident birds, respectively.

The literature search identified a total of 1244 articles, 23 of which met the selection criteria, as shown in Figure 6. As a result, we found a list $L_{3}=53$ that was sorted based on the relative ratio of sero-positives, as shown in Figure 7, as follows: 22 bird species were found most often sero-positive for WNV (with the relative ratio of sero-positives ≥ 1) and the top five species were Red-shouldered hawk, Merlin, Green heron, Eastern meadowlark and Cooper’s hawk (all with the same ratio of about 4). Conversely, 31 bird species rarely had serological evidence of exposure to WNV with American redstart having the lowest sero-prevalence.

### 3.4. Final List: $L_{f}$

The distribution of the species we identified among the lists is as follows: $L_{1}=18$ (mortality data of wild birds), $L_{2}=23$ (blood meal analysis of the *CPR*) and $L_{3}=53$ (literature review) with the number of common species, $L_{1}\cap^{\;}L_{2}=2$, $L_{1}\cap^{\;}L_{3}=7$ and $L_{2}\cap^{\;}L_{3}=20$, and $L_{1}\cap^{\;}L_{2}\cap^{\;}L_{3}=2$ species (American crow and Cooper’s hawk) belonging to all three lists. The final list of 67 bird species was obtained as, $67=\left[L_{1}+L_{2}+L_{3}-L_{1}\cap^{\;}L_{2}-L_{1}\cap^{\;}L_{3}-L_{2}\cap^{\;}L_{3}+L_{1}\cap^{\;}L_{2}\cap^{\;}L_{3}\right]=18+23+53-2-7-20+2$, representing 21% of $L_{0}=318$ species from the Avibase database [27], which includes all the species present in the Montréal area. Table 3 presents the summary results of the characteristics of each of the selected 67 bird species of interest. Characteristic variables include data on bird mortality, bird abundance, CPR feeding preference and sero-prevalence for WNV, host competence, migratory status and sites for wintering and breeding extracted from the literature.

## 4. Discussion

In this study, we constructed a priority list of bird species potentially involved in the transmission of WNV in the greater Montréal region. We constructed this list by combining three sources of data: (i) results from WNV surveillance in wild (dead) birds in the province (2002–2015); (ii) evidence from molecular blood meal analysis that selected bird species are fed upon by CPR, the primary enzootic vectors of WNV in the region, collected in mosquito surveillance in the study area in 2008 and 2014; and (iii) a literature review on evidence for exposure to WNV (sero-prevalence) and the host competence of resident bird species. There were 67 breeding species identified by these data sources, which highlights the potential complexity of WNV transmission cycles in Québec.

Out of the 67 bird species, host competences were documented for 22 bird species, including eight highly competent (competence > 1) bird species ranked as follows (from the most to the least competent): Blue jay, Common grackle, House finch, House sparrow, Song sparrow, American robin, American crow and Ring-billed gull. American crows and Blue jays were used as indicators of local WNV circulation at the beginning of the epidemics [52,53,54]. In addition, both of these species exhibit high viremia when infected [20], though mortality rates are high in both species. The mortality of infected birds shortens the overall period of virus transmission, but some infected bird species can maintain sufficiently high viremia to infect many mosquitoes during the time from disease onset to death. Not all bird species are susceptible to mortality from WVN. For example, WNV amplification in California is driven primarily by house finches, which rarely die from WNV infection, and *Culex tarsalis* as the main vector species [55].

Blood meal analysis data identified 23 bird species as potential hosts for CPR mosquitoes. Most bird species bitten by CPR mosquitoes were (from most to least preferred): Common startling, American robins, Cedar waxwing, Song sparrow and House sparrow. The preference of CPR to feed upon American robins has been reported in other studies (e.g., in [13,56]) and all species, except Cedar waxwings, are competent reservoirs for WNV in at least one study, as shown in Table 2. Bird species other than American robins likely play a role in WNV transmission, particularly as some, including some sparrow species, may have greater capacity to transmit WNV (due to longer duration and higher viremia) than American robins. 

The role of the species other than American robins in the transmission of WNV could also be related to possible transmission by competent vectors other than CPR. In eastern Canada, for example, *Aedes vexans*, a widespread mosquito, is competent to transmit WNV, but it prefers to feed upon mammals and only occasionally feeds on birds [11,57,58].

Other studies have found that House sparrows are under-represented as hosts for mosquitoes relative to their densities [13,56], while in our study they appeared over-represented. To what extent these observations may be driven by regional factors, such as climate affecting bird population densities, or the relatively urbanized nature of the study area, requires further study. Mosquitoes that were collected as part of a routine entomological WNV surveillance in CO2-baited CDC light traps [11] and gravid traps, that purport to attract greater numbers of engorged mosquitoes [59], were not used. We could not rule out the possibility that this could affect comparisons with the results of studies in which gravid traps were used, although different findings using gravid and light traps regarding blood meals have not been reported [13].

We observed a shift in feeding behavior from birds to mammals, as reported in other studies [13,60,61]. Almost all CPR blood meals were taken from birds in early summer (week 23), while the ratio of bird to mammal blood meals started to decline around mid-July. However, as pointed out by others [56] the main shift in mosquito feeding from birds to mammals occurred in parallel with the onset of reported human cases (which, in Québec, usually happens during surveillance weeks 28–31 [62]. The date of acquisition of cases reported in human-case surveillance is likely several weeks before the date of reporting in surveillance [63], so while a shift of feeding behavior from birds to mammals (including humans) may contribute to the seasonal pattern of WNV infection in humans, it is unlikely to be the main cause. 

## 5. Conclusions

These findings indicate a broad similarity in the ecology of WNV in the study region and regions in the US. We noted a similar range of key avian reservoir host species and seasonal change in host selection by mosquitoes. This work has shed light on the involvement of American robins and other bird species in the circulation of the WNV in southern Québec. However, the relative importance of some bird species as hosts of CPR and WNV in the greater Montréal area may be somewhat different to that occurring in northeastern US, and field studies are needed to confirm this and explore the consequences for the risk of WNV to the human population. In addition, studies both in the field and using modeling are necessary to elucidate the roles of each bird species, which would help to synthesize and consolidate knowledge regarding the eco-epidemiology of WNV in this area. These types of studies would allow us to improve the surveillance, control and management of WNV and possibly other vector-borne wildlife diseases, which are becoming increasingly important in North America [64,65,66].

## Figures and Tables

**Table 1:** Search strategy for the literature review.

Question	Description	Answer	
		No	Yes/Cannot Tell
Level 1: Language			
Q1	Is the paper written in English or French?	0	1
	L1 = Q1; eligible for L1 = 1		
Level 2: Title			
Q1	Does the title mention West Nile terms?	0	1
Q2	Does the title mention bird terms?	0	1
Q3	Does the title mention a region of study that is concerned (East Coast USA and Canada)?	0	1
Q4	Does the title mention terms relate to sero-prevalence, prevalence, competence, capacity or transmission?	0	1
	L2 = Q1 × Q2 (1 + Q3 + Q4); eligible for L2 ≥ 1		
Level 3: Abstract			
Q1	Does the abstract describe search results rather than a method?	0	1
Q2	Do the abstract mention terms related to bird mortality?	0	1
Q3	Do the abstract mention terms related to prevalence or sero-prevalence?	0	1
Q4	Does the abstract mention terms relate to host competence ^1^?	0	1
Q5	Does the abstract mention terms relate to host capacity ^2^?	0	1
Q6	Does the abstract specify the regions of study: states of the Eastern migratory corridor for the USA and Canada?	0	1
	L3 = Q1 × Q6 × (1 + Q2 + Q3 + Q4 + Q5); eligible for L3 ≥ 1		
Score = L1 × L2 × L3; eligible for score ≥ 1			

^1^ The host competence designates the capability of a given bird species of being infected and developing a sufficient viremia for transmitting infectious agents to vectors [39]. ^2^ The concept of host capacity brings subtlety to the skill of the host. A competent bird species can only be involved in the transmission of the virus if it is sufficiently abundant and belongs to the species on which a mosquito competent for WNV feeds [34].

**Table 2:** Results of the molecular analysis of blood meals.

Species	Family	Order	*n*	Birds (%) (*n* = 97)	Mammals (%) (*n* = 14)	Total (%) (*n* = 111) *
Birds						
American robin (*Turdus migratorius*)	Turdidae	Passeriformes	30	30.9	-	27.0
Common starling (*Sturnus vulgaris*)	Sturnidae	Passeriformes	11	11.3	-	9.9
Song sparrow (*Melospiza melodia*)	Emberizidae	Passeriformes	9	9.3	-	8.1
Cedar waxwing (*Bombycilla cedrorum*)	Bombycillidae	Passeriformes	8	8.2	-	7.2
House sparrow (*Passer domesticus*)	Passeridae	Passeriformes	7	7.2	-	6.3
Savannah sparrow (*Passerculus sandwichensis*)	Emberizidae	Passeriformes	4	4.1	-	3.6
Northern cardinal (*Cardinalis cardinalis*)	Cardinalidae	Passeriformes	3	3.1	-	2.7
Gray catbird (*Dumetella carolinensis*)	Mimidae	Passeriformes	3	3.1	-	2.7
House finch (*Haemorhous mexicanus*)	Fringillidae	Passeriformes	3	3.1	-	2.7
Black-capped chickadee (*Poecile atricapilla*)	Paridae	Passeriformes	3	3.1	-	2.7
Red-winged blackbird (*Agelaius phoeniceus*)	Icteridae	Passeriformes	2	2.1	-	1.8
Common grackle (*Quiscalus quiscula*)	Icteridae	Passeriformes	2	2.1	-	1.8
Red-eyed vireo (*Vireo olivaceus*)	Vireonidae	Passeriformes	2	2.1	-	1.8
Cooper’s hawk (*Accipiter cooperii*)	Accipitridae	Accipitriformes	1	1.0	-	0.9
Green heron (*Butorides virescens*)	Ardeidae	Pelecaniformes	1	1.0	-	0.9
Northern flicker (*Colaptes auratus*)	Picidae	Piciformes	1	1.0	-	0.9
American crow (*Corvus brachyrhynchos*)	Corvidae	Passeriformes	1	1.0	-	0.9
American yellow warbler (*Dendroica petechia*)	Parulidae	Passeriformes	1	1.0	-	0.9
Ovenbird (*Seiurus aurocapilla*)	Parulidae	Passeriformes	1	1.0	-	0.9
American goldfinch (*Spinus tristis*)	Fringillidae	Passeriformes	1	1.0	-	0.9
Chipping sparrow (*Spizella passerina*)	Emberizidae	Passeriformes	1	1.0	-	0.9
Warbling vireo (*Vireo gilvus*)	Vireonidae	Passeriformes	1	1.0	-	0.9
Mourning dove (*Zenaida macroura*)	Columbidae	Passeriformes	1	1.0	-	0.9
Mammals						
White-tailed deer (*Odocoileus virginianus*)	Cervidae	Artiodactyla	7	-	50.0	6.3
Bovine (*Bos taurus*)	Bovidae	Artiodactyla	2	-	14.3	1.8
Cat (*Felis catus*)	Felidae	Carnivora	2	-	14.3	1.8
Human (*Homo sapiens*)	Hominidae	Primates	2	-	14.3	1.8
Mule deer (*Odocoileus hemionus*)	Cervidae	Artiodactyla	1	-	7.1	0.9

* of the 273 blood meal samples analyzed, 111 resulted in interpretable sequencing while 162 did not.

**Table 3:** Characteristics of bird species affected by WNV in the Montréal area.

Family	Species (English Name)	Species (Latin Name)	Literature			Mortality, % (*n*) ^3^		$\mathrm{Abundance}{\;}^{4}a_{s}$	$\mathrm{CPRfeeding}{\;}^{5}f_{s}$	Status ^6^	Wintering Area^7^	Breeding Area ^7^	Spring Migration
			Seroprevalence (%) ^1^ Min (*n*)–Max (*n*)	Host Competence ^2^ Min-Max		2001–2008	2009–2017						
Anatidae	Canada Goose	*Branta canadensis*	0.8 (1038)–29 (7)		0.03–0.05					M, R	S	QC, N	March
Anatidae	Wood Duck	*Aix sponsa*	0 (1)–2.5 (12)				0 (1)			M, R	S	QC, N, S	March
Anatidae	Mallard ^†^	*Anas platyrhynchos*	8 (13)–10.6 (66)		0.48–0.5		50 (4)			M, R	S	QC, N, S	March
Anatidae	Ring-necked Duck	*Aythya collaris*	5.3 (19)							M, R	S	QC, N	March
Ardeidae	Green Heron	*Butorides virescens*	100 (1)					0.0049	0.0103	M	S	QC, N, S	April
Accipitridae	Northern Harrier†	*Circus cyaneus*					100 (1)			M	QC, N, S	QC, N	March
Accipitridae	Sharp-shinned Hawk†	*Accipiter striatus*					86.4 (22)			M, R	QC, N, S	QC, N, S	March
Accipitridae	Cooper’s Hawk†	*Accipiter cooperii*	100 (1)				65.4 (26)	0.0036	0.0103	M, R	QC, N, S	QC, N, S	March
Accipitridae	Northern Goshawk†	*Accipiter gentilis*					85.7 (7)			M, R	QC, N	QC, N	March
Accipitridae	Bald Eagle†	*Haliaeetus leucophalus*					25 (4)			M	N, S	QC, N, S	March
Accipitridae	Red-shouldered Hawk†	*Buteo lineatus*	100 (1)				100 (5)			M, R	QC, N, S	QC, N, S	March
Accipitridae	Broad-winged Hawk†	*Buteo platypterus*					70 (10)			M	S	QC, N, S	April
Accipitridae	Red-tailed Hawk†	*Buteo jamaicensis*					76.9 (13)	0.0013		M, R	QC, N, S	QC, N, S	March
Charadriidae	Killdeer	*Charadrius vociferus*			0.84–0.87					M	N, S	QC, N, S	March
Laridae	Ring-billed Gull	*Larus delawarensis*			1.18–1.26		0 (3)			M, R	QC, N, S	QC, N	February
Columbidae	Rock Pigeon	*Columba livia*	4.3 (23)–55 (20)		0					R	QC, N, S	QC, N, S	
Columbidae	Mourning Dove	*Zenaida macroura*	3.8 (26)–57.69 (26)		0–0.19		0 (2)	0.0137	0.0103	M, R	QC, N, S	QC, N, S	April
Cuculidae	Yellow-billed Cuckoo	*Coccyzus americanus*	5.9 (17)–100 (1)							M		QC, N, S	Mai
Strigidae	Great Horned Owl†	*Bubo virginianus*	44.4 (9)		0.68–0.9		50 (6)			R	QC, N, S	QC, N, S	
Strigidae	Snowy Owl†	*Bubo scandiacus*					100 (2)			M	QC, N		March
Strigidae	Barred Owl†	*Strix varia*					66.7 (3)			R	QC, N, S	QC, N, S	
Strigidae	Northern Saw-whet Owl†	*Aegolius acadicus*					50 (8)			R	QC, N	QC, N	
Picidae	Hairy Woodpecker	*Picoides villosus*	0 (14)–14.3 (7)							R	QC, N, S	QC, N, S	
Picidae	Northern Flicker	*Colaptes auratus*			0.06–0.14			0.0178	0.0103	M, R	N, S	QC, N, S	April
Picidae	Pileated Woodpecker	*Dryocopus pileatus*	20 (5)							R	QC, N, S	QC, N, S	
Falconidae	American Kestrel	*Falco sparverius*	16 (152)–100 (1)		0.79–0.93	0 (1)	0 (5)			M, R	QC, N, S	QC, N, S	April
Falconidae	Merlin†	*Falco columbarius*	100 (1)				65.5 (55)			M, R	QC, N, S	QC, N	March
Falconidae	Gyrfalcon†	*Falco rusticolus*					100 (1)			M, R	QC		
Tyrannidae	Great Crested Flycatcher	*Myiarchus crinitus*	2 (50)							M		QC, N, S	May
Vireonidae	Warbling vireo	*Vireo gilvus*						0.0189	0.0103	M		QC, N, S	May
Vireonidae	Red-eyed vireo	*Vireo olivaceus*						0.0288	0.0206	M		QC, N, S	May
Corvidae	Blue Jay†	*Cyanocitta cristata*	0.8 (121)–35.8 (134)		2.39–2.55	23.6 (886)	66.7 (6)	0.0093		R	QC, N, S	QC, N, S	
Corvidae	American Crow†	*Corvus brachyrhynchos*	3.2 (157)–68.3 (183)		1.04–1.62	40.1 (1418)	73.9 (46)	0.0549	0.0103	R	QC, N, S	QC, N, S	
Hirundinidae	Tree Swallow	*Tachycineta bicolor*	2.6 (156)							M	S	QC, N, S	March
Paridae	Black-capped Chickadee	*Poecile atricapillus*	0 (107)					0.0511	0.0309	R	QC, N	QC, N	
Sittidae	White-breasted Nuthatch	*Sitta carolinensis*	0 (40)–2.9 (35)							R	QC, N, S	QC, N, S	
Troglodytidae	House Wren	*Troglodytes aedon*	5.9 (17)							M	S	QC, N, S	April
Turdidae	Eastern Bluebird	*Sialia sialis*	2.4 (126)							M, R	N. S	QC, N, S	March
Turdidae	Swainson’s Thrush	*Catharus ustulatus*	2.13 (47)–3.1 (32)							M		QC, N	May
Turdidae	Wood Thrush	*Hylocichla mustelina*	1 (101)–15.6 (32)							M		QC, N, S	May
Turdidae	American Robin	*Turdus migratorius*	2.6 (76)–10.11 (366)		1.04–1.1			0.0578	0.3093	M, R	QC, N, S	QC, N, S	April
Mimidae	Gray Catbird	*Dumetella carolinensis*	3.5 (2706)–35 (17)		0.07–0.1			0.0158	0.0309	M, R	N, S	QC, N, S	May
Mimidae	Brown Thrasher	*Toxostoma rufum*	3.7 (643)–10.5 (19)							M, R	N, S	QC, N, S	April
Sturnidae	Common Starling	*Sturnus vulgaris*			0.16–0.22			0.2045	0.1134	R	QC, N, S	QC, N, S	
Bombycillidae	Cedar Waxwing	*Bombycilla cedrorum*	20 (5)					0.0671	0.0825	M, R	QC, N, S	QC, N, S	March
Parulidae	Ovenbird	*Seiurus aurocapilla*	0.9 (115)–3.1 (32)					0.0024	0.0103	M	S	QC, N, S	May
Parulidae	Common Yellowthroat	*Geothlypis trichas*	0.7 (299)							M	S	QC, N, S	May
Parulidae	American Redstart	*Setophaga ruticilla*	0.4 (280)							M	S	QC, N, S	May
Parulidae	Cape May Warbler †	*Setophaga tigrina*					100 (1)			M		QC, N	May
Parulidae	Northern Parula	*Setophaga americana*	4.7 (43)							M	S	QC, N, S	May
Parulidae	Yellow Warbler	*Setophaga petechia*			1			0.0368	0.0103	M		QC, N, S	April
Passerellidae	Chipping Sparrow	*Spizella passerina*	3 (59)					0.0652	0.0103	M, R	S	QC, N, S	April
Passerellidae	Savannah Sparrow	*Passerculus sandwicheris*						0.0021	0.0412	M	S	QC, N	April
Passerellidae	Song Sparrow	*Melospiza melodia*	0 (13)–3.4 (88)		1.2			0.0431	0.0928	M, R	QC, N, S	QC, N, S	April
Passerellidae	Eastern Towhee	*Pipilo erythrophthalmus*	0.7 (144)–9.6 (197)							M	N, S	QC, N, S	April
Cardinalidae	Scarlet Tanager	*Piranga olivacea*	2.8 (71)							M		QC, N, S	April
Cardinalidae	Northern Cardinal	*Cardinalis cardinalis*	6.2 (503)–52.2 (115)		0.38			0.0313	0.0309	R	QC, N, S	QC, N, S	
Cardinalidae	Rose-breasted Grosbeak	*Pheucticus ludovicianus*	1 (98)–5 (22)							M		QC, N	April
Cardinalidae	Indigo Bunting	*Passerina cyanea*	3.6 (28)–2.2 (223)							M	S	QC, N, S	April
Icteridae	Eastern Meadowlark	*Sturnella magna*	100 (1)							M	N, S	QC, N, S	March
Icteridae	Baltimore Oriole	*Icterus galbula*	8.3 (12)							M	S	QC, N, S	May
Icteridae	Red-winged Blackbird	*Agelaius phoeniceus*	0 (63)–10.5 (67)		0.9–0.99			0.1036	0.0206	M, R	QC, N, S	QC, N, S	March
Icteridae	Brown-headed Cowbird	*Molothrus ater*	1.8 (494)–12.5 (24)		0					M, R	QC, N, S	QC, N	April
Icteridae	Common Grackle	*Quiscalus quiscula*	0 (106)–15.4 (13)		1.39–2.04			0.0462	0.0206	M, R	QC, N, S	QC, N, S	March
Fringillidae	House Finch	*Haemorhous mexicanus*	2 (927)–100 (5)		1.29–1.8			0.0125	0.0309	M, R	QC, N, S	QC, N, S	March
Fringillidae	American Goldfinch	*Spinus tristis*	0.3 (337)–3.1 (128)					0.0666	0.0103	M, R	QC, N, S	QC, N, S	February
Passeridae	House Sparrow	*Passer domesticus*	1.6 (1042)–51 (107)		1.25–1.6		0 (1)	0.0508	0.0722	R	QC, N, S	QC, N, S	

Species = bird species for which blood meal data are available in our study area (*n* = 20); † = bird species for which mortality data are available in available study area (*n* = 18); ^1^ Minimum and maximum percentages of WNV sero-positive birds (*n* = sample size) [6,7,18,40,41,42,43,44,45,46,47,48,49]; ^2^ Minimum and maximum index of host competence for WNV [20,50,51]; ^3^ Percentage of dead birds positive for WNV (*n* = sample size) (CWHC). Data used in the construction of list *L_1_*; ^4^ $a_{s}$ is the density of species *s* divided by the total density of the avian community—data come from the EPOQ database. The sum of all $a_{s}$ is equal to one; ^5^ *f_i_* is the fraction of total blood meals taken by *Cx. pipiens—restuans* from host *s* [9]. The sum of all *f_i_* is equal to one; ^6^ M: Migratory bird species; R: Resident bird species; ^7^ S: South USA; N: North USA; QC: Québec.
